# The Comprehensive Biomechanics and Load-Sharing of Semirigid PEEK and Semirigid Posterior Dynamic Stabilization Systems

**DOI:** 10.1155/2013/745610

**Published:** 2013-08-04

**Authors:** D. K. Sengupta, Brandon Bucklen, Paul C. McAfee, Jeff Nichols, Raghavendra Angara, Saif Khalil

**Affiliations:** ^1^Dartmouth-Hitchcock Medical Center, One Medical Center Drive, Lebanon, NH 03756, USA; ^2^Globus Medical, Inc., Valley Forge Business Center, 2560 General Armistead Avenue, Audubon, PA 19403, USA; ^3^Towson Orthopaedic Associates, P.A.—Scoliosis and Spine Center, O'Dea Medical Arts Building, Suite 104, 7505 Osler Drive, Towson, MD 21204, USA

## Abstract

Alternatives to conventional rigid fusion have been proposed for several conditions related to degenerative disc disease when nonoperative treatment has failed. Semirigid fixation, in the form of dynamic stabilization or PEEK rods, is expected to provide compression under loading as well as an intermediate level of stabilization. This study systematically examines both the load-sharing characteristics and kinematics of these two devices compared to the standard of internal rigid fixators. Load-sharing was studied by using digital pressure films inserted between an artificially machined disc and two loading fixtures. Rigid rods, PEEK rods, and the dynamic stabilization system were inserted posteriorly for stabilization. The kinematics were quantified on ten, human, cadaver lumbosacral spines (L3-S1) which were tested under a pure bending moment, in flexion-extension, lateral bending, and axial rotation. The magnitude of load transmission through the anterior column was significantly greater with the dynamic device compared to PEEK rods and rigid rods. The contact pressures were distributed more uniformly, throughout the disc with the dynamic stabilization devices, and had smaller maximum point-loading (pressures) on any particular point within the disc. Kinematically, the motion was reduced by both semirigid devices similarly in all directions, with slight rigidity imparted by a lateral interbody device.

## 1. Introduction

Conventional instrumentation to achieve fusion in the lumbar spine utilizes rigid rods and pedicle screws [1–3]. Rigid rod fixation is criticized to reduce load-sharing and inhibit fusion mass formation because of the stress-shielding effect [4]. Not only does load-sharing influence fusion, but it may also affect a patient's pain level, adjacent segment kinematics, and the potential for device failure following spine surgery [2]. Semirigid instrumentations, such as polyetheretherketone (PEEK) rods and titanium rods with helical grooves, are designed to increase load-sharing in attempt to induce compression on the bone graft and promote bone remodeling as first credited by Wolff [4]. There is certainly evidence of osteoblastic response to mechanical activation, in various forms, as well as increases in bone formation rate following bouts of loading [5, 6]. Semirigid PEEK instrumentation attempts to allow loading through the anterior column, but most studies show the stiffness of these constructs to be relatively high [7].

One hypothesized benefit of a dynamic device is to restore the loading of the damaged disc to similar thresholds as a normal disc would tolerate. For example, with damage comes reduced proteoglycan content, overload of the annulus, change in homogeneity of material properties, depressurization of the nucleus, and inability of the disc to hydrophilically retain water [3, 8]. Normal physiological loads on a damaged disc will produce stress concentrations higher and more disparate than a corresponding healthy disc. Therefore, one goal of semirigid stabilization should be to reduce and redistribute these loads off the disc to some degree, while trying to maintain a similar loading as what a healthy disc would experience, all in the presence of fusion.

Dynamic stabilization systems may be used as a load-sharing device, to mechanically stimulate bone cells toward fusion. The purpose of the device is to act as semirigid instrumentation, with the ability to dampen the loads as the spine moves. Design of posterior dynamic stabilization devices (PDSs) is very difficult for the reason that every diseased disc is not diseased in the same way and to the same extent as one another. How much offloading is ideal remains a matter of debate. Sengupta et al. suggest that pain alleviation is determined by the uniformity of loading on the disc space, as a fully healthy disc is expected to act as a uniform load bearing structure [3, 8].

In this study, the authors are interested in evaluating the load-sharing and kinematic properties of the spectrum of surgical options, from rigid, using titanium rods, to semirigid, using PEEK rods, to PDS, using polymer-based posterior dampeners. The goal was to quantify load-sharing as well as to determine the distribution of these loads within the interbody space. 

## 2. Materials and Methods

There are two arms to this study. The first arm is determining the load-sharing through the anterior column as determined by the type of posterior instrumentation. The second arm is determining the kinematic range-of-motion as determined by the type of posterior instrumentation. Due to difficulty in measuring regional loading through an intervertebral disc *in vivo* or *in vitro*, mechanical testing was conducted on a spine model with a flat disc surface, as to accommodate a pressure sensor on the disc. The range-of-motion characterization was carried out on human cadaveric spines.

### 2.1. Load-Sharing

#### 2.1.1. Testing Fixtures and Protocol

Tests were performed on lumbar spine models, prepared according to the ASTM F1717 standard corpectomy model (modified with a construct height of 28 mm from top screw to bottom screw) using ultrahigh molecular weight polyethylene (UHMWPE) blocks. The load-sharing of the vertebral disc was simulated through controlled mechanical testing using a MTS Bionix Servohydraulic Test System (Eden Prairie, MN) with an MTS 661.18 Force Transducer, 2.5 kN maximum. Prior to testing, each construct was soaked in a saline (0.9%) bath at body temperature (37°C) for 1 hour. An axial compressive bending load was applied to the construct at a rate of 10 mm/min in ambient air until 320 N was reached. The moment arm was chosen so that the bending moment, with respect to the posterior instrumentation, in maximum flexion was 12.8 Nm resulting in a 0.04 m moment arm. The constructs were subjected to cyclical loading for three cycles, and the data were captured from the third cycle.

The anterior column load was measured using Tekscan pressure mapping software (I-Scan Pressure Measurement System, Boston, MA) and a digital pressure film (Model 5051) placed between the endplate of the polyethylene vertebral body and the modified interbody spacer device (CONTINENTAL, Globus Medical, Audubon, PA) to detect contact pressures. The pressure film consists of a square grid of 44 × 44 sensor cells with a row and column width of 0.03 inches. Each cell registers the force passing through it. Based on the known area of each cell, the software postprocesses pressure as well as other parameters involving regional data averaging.

**Figure 1:**
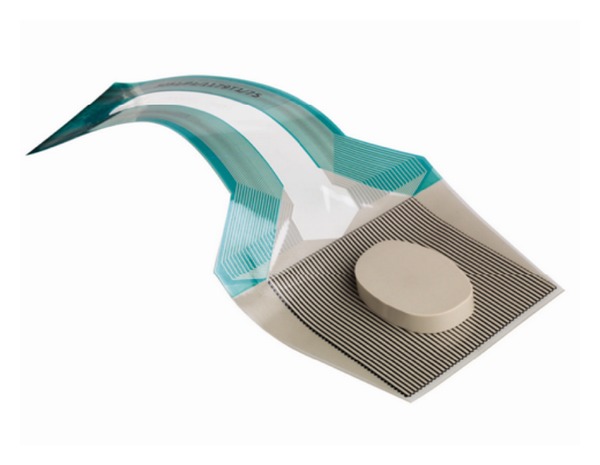
Tekscan pressure film used to measure contact pressures on the spacer. Note that during testing the film was placed above the spacer.

**Figure 2:**
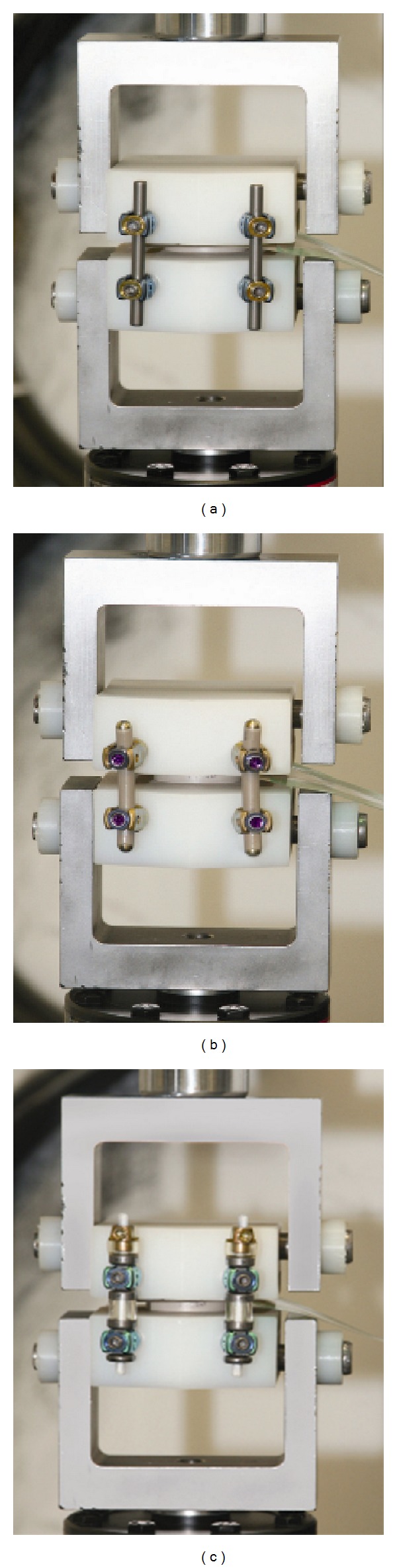
Testing constructs loaded in fixtures. Left to right: rigid rods, PEEK rods, and posterior dynamic stabilization. The pressure film can be seen above the spacer.

The spacer was machined from PEEK, and two windows (in the shape of bone graft windows) were milled to a depth of 3 mm on the bottom surface (to ensure consistent location), while the top surface of the spacer remained flat (test surface) (Figure 1). The top surface of the spacer was tested via a profilometer to ensure no preexisting roughness which could cause pressure artifacts. Solid rigid polyurethane foam (Sawbones, density 20 pcf, Vashon, WA) which contained a boss of the same window shape as the spacer was inserted into the bottom test block (UHMWPE). The windows were used to secure a press fit of the spacer onto the bottom polyurethane foam insert, so that the spacer would not move throughout the testing. A flat polyurethane foam insert was used in the upper test block (UHMWPE). The sequence of load transfer was therefore through the MTS machine, upper test block, upper polyurethane foam insert, pressure film, spacer, lower polyurethane foam insert, and lower test block. The pressure film was placed between the spacer and upper polyurethane foam block, according to Figure 2. A fixed clearance was introduced to ensure consistency in the location of first contact. The clearance between the interbody spacer and upper test fixture was chosen at 400 *μ*m and was measured on all four sides, prior to testing.

#### 2.1.2. Posterior Stabilization

**Figure 3:**
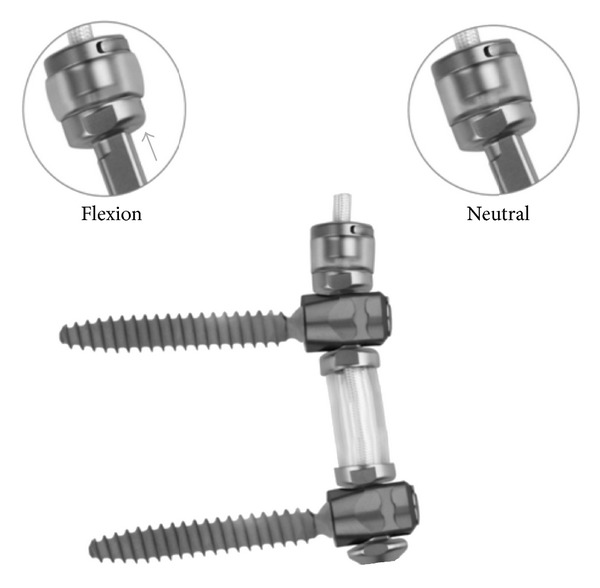
The TRANSITION Stabilization System. The cephalad bumper shown in neutral and flexed positions.

Three different posterior instrumentation systems representing rigid (titanium rods, REVERE, Globus Medical, Audubon, PA), semirigid (PEEK rods, LEGACY, Medtronic, Memphis, TN), and semirigid (dynamic stabilization, TRANSITION, Globus Medical, Audubon, PA) stabilization methods were tested. The pedicle screws were placed with the tulip heads nearly flush with respect to the UHMWPE test blocks with just enough space for polyaxial head toggle. The PDS device, TRANSITION Stabilization System, was evaluated (Figure 3), and it requires some explanation. The device consists of titanium spools over a polyethylene terephthalate (PET) cord which are dropped in between pedicle screws of adjacent levels. A polycarbonate urethane (PCU) polymer spacer surrounds the cord and fits between the spools which is used to buffer compressive forces. The PCU spacer between the pedicle screws is compressible, to allow normal extension with a soft end point, and the PCU bumper is compressible, allowing a dampened flexion motion. A PET cord which is not attached directly to the pedicle screw head but imbedded within titanium spools allows small interpedicular distance (IPD) changes.

Prior to testing, the pressure mapping system was calibrated using loads of 50% and 100% of the maximum load (320 N) without posterior instrumentation in place, in order to correlate the raw sensor readings to standardized pressure units (pounds per square inch). Five samples were tested. Each sample was prepared by assembling the posterior instrumentation on the test fixtures with appropriate test clearance. Following testing, the assembly was deconstructed, soaked in saline, and subsequently reconstructed with the next sample. Data was recorded from the pressure film in real time during the three loading and unloading cycles and saved as a sequence of image files. Data analysis and postprocessing was completed on the pressure profile of the image slice corresponding to the maximum load of 320 N for the last cycle.

#### 2.1.3. Data Analysis

**Figure 4:**
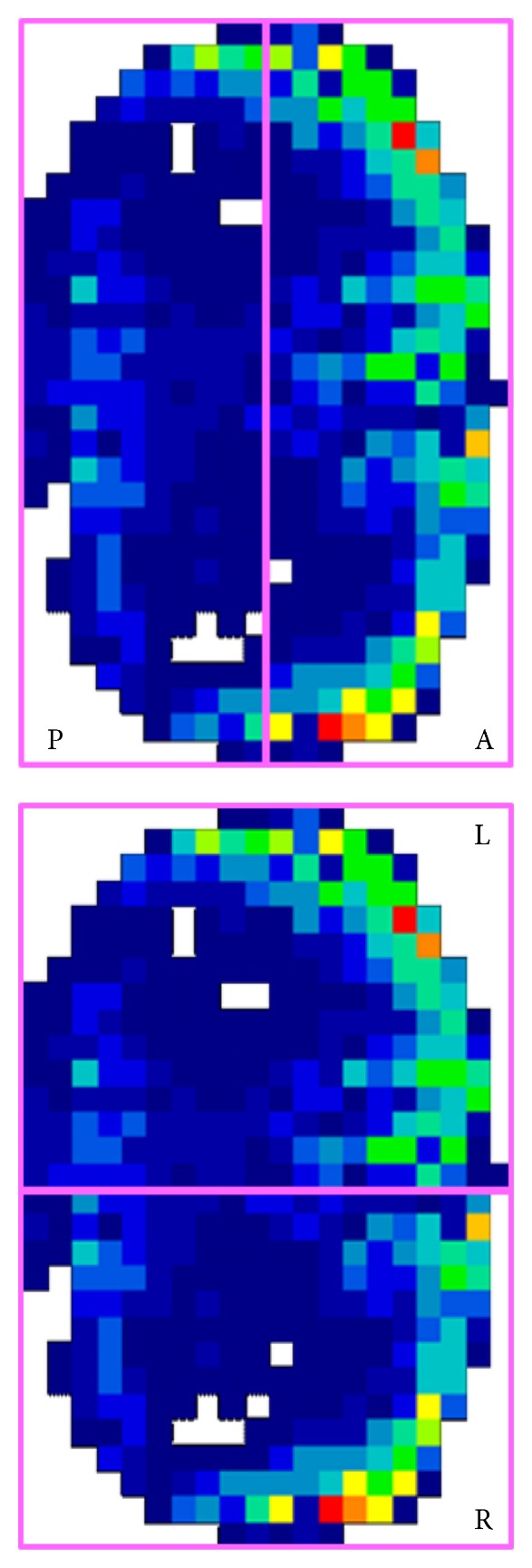
Pressure area division by bounding box: anterior (A), posterior (P), left (L), and right (R).

The total force passing through the spacer (i.e., pressure film) was used to estimate load-sharing between the anterior and posterior column. The load passing through the spacer represented anterior column load-sharing, while the remaining load was assumed to pass through the posterior instrumentation and represented posterior column loading. Subsequent analysis involved splitting the anterior column spacer area into four distinct regions, hereafter referred to as bounding boxes: anterior, posterior, left, and right, according to the symmetrical midlines of the spacer itself (see Figure 4). For each bounding box, the following quantities were tabulated: total force, box pressure, and peak (maximum) box pressure. The total force is the sum of the forces in the rectangular bounding box. Box pressure is the sum of the forces in the rectangular bounding box divided by the area of the bounding box. Peak box pressure is the maximum localized pressure in that bounding box. Statistical comparisons were made using two-tailed student's *t*-tests assuming equal variance, with a probability of type I error, *α* = 0.05 (*n* = 5).

### 2.2. Kinematics

#### 2.2.1. Specimen Preparation and Test Constructs

**Figure 5:**
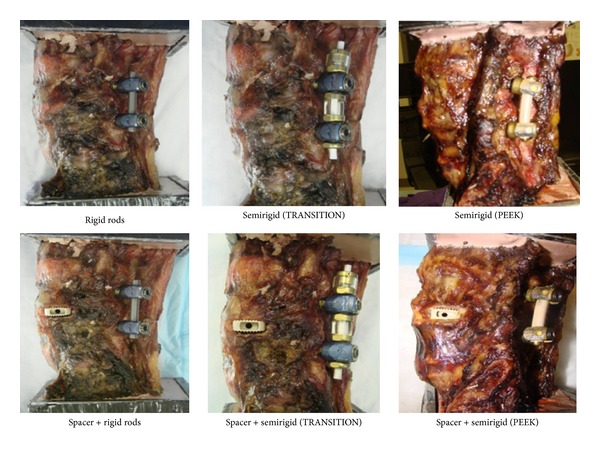
Select test images from flexibility testing, showing rigid and semirigid devices.

**Figure 6:**
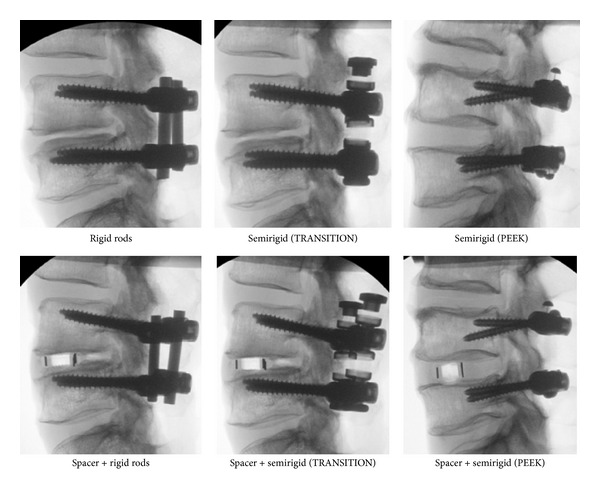
Select radiographs from flexibility testing, showing rigid and semirigid devices.

Ten human cadaver lumbosacral spines (L3-S1) were tested under a pure moment of 7 N-m, using a 6-degree-of-freedom spine tester in flexion-extension (FE), lateral bending (LB), and axial rotation (AR). The spines were fixed proximally at L3 and distally at S1 in a three-to-one mixture of Bond Auto Body Filler and fiberglass resin (Bondo Mar-Hyde Corp., Atlanta, GA). The specimens were divided into two groups of five, according to the type of semirigid fixation applied. Each type of transpedicular semirigid instrumentation utilized pedicle screws of a different design and could not be reused on the same specimen without sacrificing screw purchase. Posterior fixation was tested with and without a lateral interbody spacer (TransContinental, Globus Medical) and in the injured state following a lateral discectomy (Figures 5 and 6). Results are presented as a percentage of intact range-of-motion (ROM).

#### 2.2.2. Test Setup and Data Analysis

The spine was affixed to a six-degree-of-freedom (6DOF) testing apparatus via magnetization, and pure unconstrained bending moments will be applied in the physiologic planes of the spine at room temperature using a multidirectional hybrid flexibility protocol [9]. The 6DOF machine applies unconstrained loading through three cephalad stepper motors placed in each of the three physiological rotation axes. Moreover, the supports are mounted on air bearings to provide near frictionless resistance to the natural kinematics of the spine. Plexiglass markers, each having three infrared light-emitting diodes, were secured rigidly to each vertebral body via bone screws to track its motion with the Optotrak Certus (NDI, Inc., Waterloo, Canada) motion analysis system. The location of the markers (denoting a rigid body) were aligned approximately sagitally along the curvature of the spine. The Optotrak Certus software superimposes the coordinate systems of two adjacent vertebral bodies in order to inferentially determine the relative Eularian rotations in each of the three planes.

Data analysis was conducted using independent single factor ANOVA, with both groups combined. The data was first normalized to intact motion and underwent a log transformation to remove unequal variances. The Student-Newman-Keuls post hoc test was applied to all eight comparison groups at a level-1 alpha value of 0.05.

## 3. Results

### 3.1. Load-Sharing

**Figure 7:**
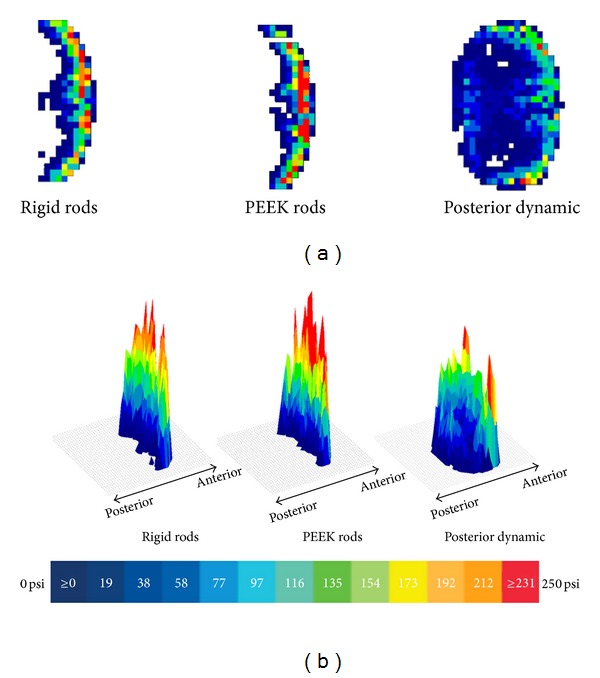
Example of pressure film data showing pressure contact area of spacer with blue colors representing low pressures and red colors representing high pressures, in two dimensions (a) and three dimensions (b) for each posterior instrumentation type.

A representative example of the recorded data is shown in Figure 7 (during maximum applied loading) for each type of posterior fixation. TekScan software postprocesses the output of each sensor cell into color-coded regions from low to high for easy visualization. A three-dimensional view is useful to assess the distribution and locations of maximum pressure.

**Figure 8:**
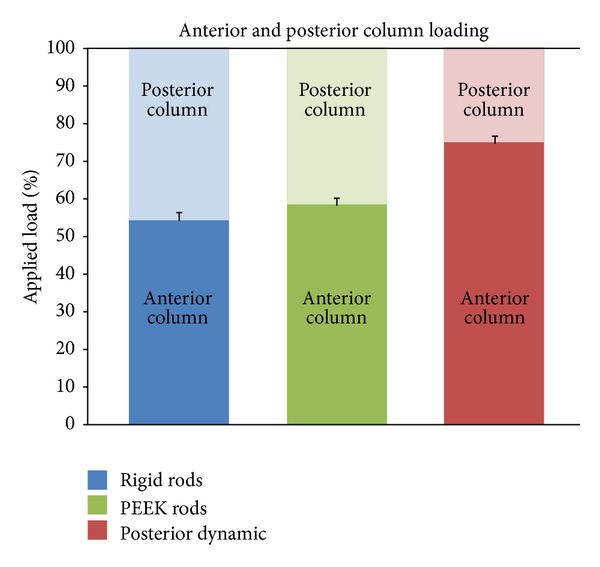
Anterior and posterior columns load-sharing. The bars represent standard deviations in measured anterior column load.

The force through the entire spacer was used to determine the percentage of anterior and posterior columns loading as a fraction of the applied load (Figure 8). All load not passing through the pressure film (located in the anterior column) was considered to pass through the posterior instrumentation (located in the posterior column). No energy dissipation due to friction or any form was considered. Anterior column load-sharing was 55%, 59%, and 75%, for rigid rods, PEEK rods, and posterior dynamic stabilization, respectively. The posterior dynamic stabilization system transferred statistically more load than rigid or PEEK rods. Rigid and PEEK rods did not statistically differ from each other in their ability to transfer load through the anterior column. Moreover, of the three instrumentation types tested, the dynamic stabilization most closely approximated the load-sharing of the intact lumbar spine as an 80%–20% distribution in the anterior-posterior column as described by White and Panjabi, 1990 [10]. It should be noted that posterior elements and their contribution toward the load-sharing could not be included in the mechanical model.

#### 3.1.1. Regional Loading

**Figure 9:**
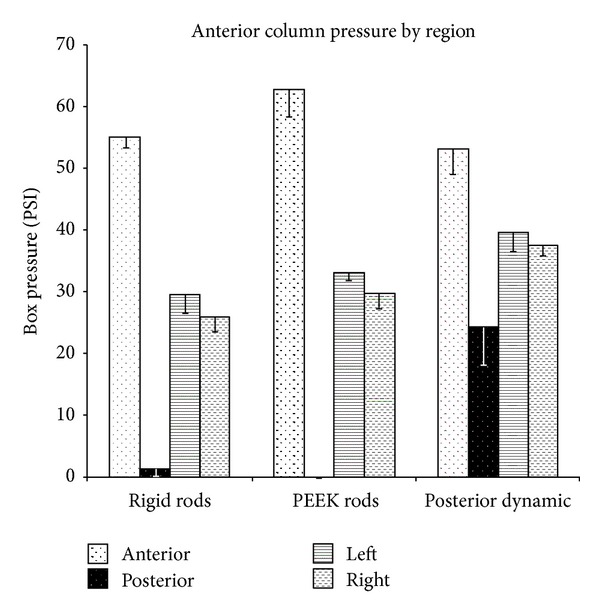
Average box pressure of the anterior, posterior, left, and right portions of the spacer as measured through the pressure film (according to Figure 4).

**Figure 10:**
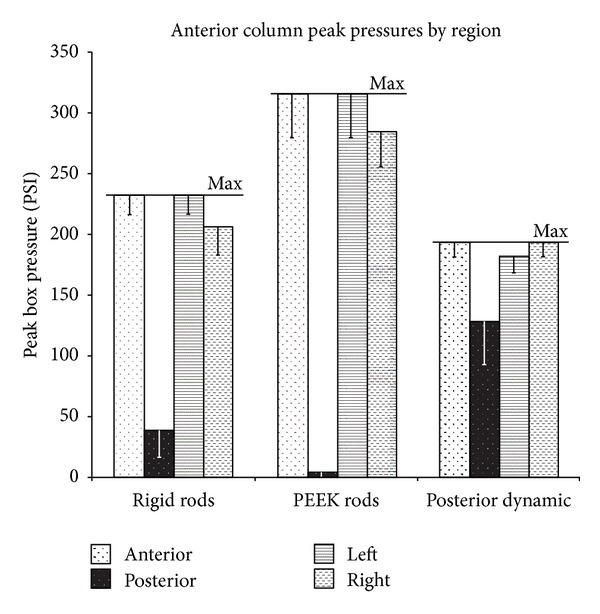
Peak (or maximum) box pressure of the anterior, posterior, left, and right portions of the spacer as measured through the pressure film (according to Figure 4).

The spacer area was divided into bounding boxes describing anterior, posterior, left, and right regions. The average (Figure 9) and peak (Figure 10) pressures in each bounding box are reported. The reader should note the distinction between anterior and posterior bounding boxes (Figure 4) and the anterior and posterior columns loading as described previously.

In all types of fixation, the average anterior box pressure was larger than the posterior box pressure, and left and right pressures were similar (Figure 9). The disparity between anterior and posterior pressures was largest for PEEK rod instrumentation (63 PSI) and smallest for posterior dynamic instrumentation (29 PSI). The regional pressure profile created by PEEK rods was similar to that of rigid rods, except that PEEK had a higher anterior pressure. In all cases, posterior dynamic stabilization resulted in statistically higher average pressure readings, in the posterior, left, and right sides of the spacer, than the other posterior fixation groups. The PEEK rods were the only constructs to result in a statistical difference in left versus right box pressures.

In all types of fixation, the peak anterior box pressure was larger than the peak posterior box pressure, and there were no statistical differences between left and right pressures (Figure 10). The disparity between anterior and posterior peak pressures was largest for PEEK rod instrumentation (312 PSI) and smallest for posterior dynamic instrumentation (65 PSI). The maximum pressure across the entire spacer (denoted by “max” in Figure 10) was the largest for PEEK rods (316 PSI), followed by rigid rods (233 PSI), and smallest for posterior dynamic instrumentation (193 PSI). All three instrumentation types were statistically different.

### 3.2. Kinematics

**Figure 11:**
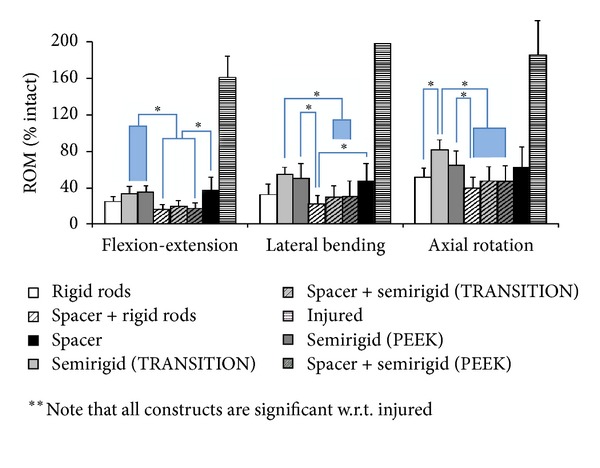
The biomechanical flexibility results normalized to intact motion (100%) by mode. Statistical indicators are shown with asterisks.

As per Figure 11, in the without-interbody group, rigid rods achieved the highest level of fixation (FE: 25%, LB: 33%, AR: 52%), with both semirigid systems demonstrating equivalence (TRANSITION; FE: 34%, LB: 54%, AR: 82%; PEEK Rods; FE: 35%, LB: 51%, AR: 65%). The addition of a lateral interbody spacer provided much stability, similar to that of semirigid instrumentation without interbody, in all three loading modes. In the interbody group, rigid rods achieved the highest level of fixation (FE: 16%, LB: 23%, AR: 40%) compared to the semirigid systems (TRANSITION FE: 20%, LB: 30%, AR: 48%; PEEK Rods FE: 18%, LB: 30%, AR: 47%). Semirigid systems led to gradual decrease of stiffness of 10%, 20%, and 20% in FE, LB, and AR, respectively, when compared to rigid systems without interbody. With the addition of a lateral interbody device, range-of-motion of semirigid systems was reduced by 16%, 22%, and 26%. There were no detectable differences between the semirigid devices tested. 

## 4. Discussion

The stiffness of the spine after surgery is a combination of the applied instrumentation and fusion mass. With difficulty in predicting the contribution of the fusion mass, this study investigated the rigidity of posterior instrumentation imparted on the spine and the allowable anterior load-sharing. The benefits of semirigid systems may further contribute to the stiffness of the fusion mass, but could not be evaluated.

The hypothesis was that anterior column load-sharing would be higher for posterior dynamic stabilization (PDS) and PEEK rods than rigid systems. According to Sengupta et al., load-sharing and uniformity of loading are two important factors responsible for mechanical back pain, when occurring abnormally [3, 8]. Load-sharing at the index level is useful to promote fusion by stimulating bone cells to form new bone in the graft space. Most patients complain of acute or short bouts of pain, which could be triggered with peak contact pressures (abnormal load-sharing) or nonuniform pressures on a compromised disc. Load-sharing influences adjacent level motion, which has been considered as contributing to adjacent level disease. While previous biomechanical studies have described the relationship between adjacent level problems and kinematic behavior of PDS devices, there is no reported study that investigated load-sharing, which may prove to be clinically as important as global range-of-motion—though consistent clinical evidence is even lacking for motion [11, 12]. In this study, anterior column load-sharing was improved by the use of a semirigid posterior dynamic stabilization device when compared to semirigid PEEK or rigid fixation. The anterior-posterior column distribution was 55%–45%, 59%–41%, and 75%–25%, for rigid rods, PEEK rods, and posterior dynamic stabilization, respectively. The PDS device approximated the 80%–20% distribution of the normal spine as outlined by White and Panjabi, 1990 [10]. No statistical difference in anterior column loading existed between PEEK or rigid rods, but the PDS device provided statistically more load through the anterior column than both systems.

The load distribution across the interbody spacer area proved to be more uniform with posterior dynamic stabilization when compared to semirigid PEEK or rigid fixation as evidenced by Figures 5, 7, and 8. With rigid and PEEK rods, only nominal load was transferred to the posterior aspects of the spacer. PEEK rods consistently demonstrated larger average and maximum pressures on the anterior region of the spacer, when compared to PDS or rigid fixation. Dynamic stabilization and rigid rods have similar pressures on the anterior region of the spacer but differ dramatically in the posterior, left, and right portions of the spacer, which are much more uniform and statistically higher for PDS when compared to rigid rods. It should be noted, due to the predefined clearance of 400 *μ*m, that load was first transmitted to the anterior portion of the spacer, to ensure consistency of testing.

Another difference between the PDS design and conventional designs is that the PDS device in this study utilized a PET cord which was not attached directly to the pedicle screw head but imbedded within titanium spools which allow some travel or sliding to compress and engage the soft bumper. This effect may have helped to redistribute the loads. This redistribution seems very evident in Figure 5, where the PDS device shows more coverage and symmetry in loading. Additionally, the repeatability of the testing was high across all five samples.

Peak or maximum pressures are of great concern and may be mechanically induced pain generators even during normal loading. Ideally, a PDS device would reduce abnormally high pressures as well as reduce disparities between different portions of the interbody space. While total load-sharing with the PEEK device is marginally improved compared to rigid rods, the disparity between anterior and posterior regions of PEEK is the least favorable, with a 312 psi difference. The PDS device has a more favorable profile of maximum pressures which are lower in magnitude and more equally distributed across different regions (largest regional difference 65 psi). Statistically speaking, PEEK rods produced the highest maximum pressures, while the PDS device produced the least.

There are very few literature studies which looked at load-sharing in posterior dynamic stabilization devices, of which the data from the current study could be compared. Analytical finite element studies on L3-L4 spinal segment showed that axial forces across the anterior column would be 29%, 67%, and 59% of the applied loading for rigid rods, PEEK rods, and PDS, respectively^4^. However, the PDS device examined was a nitinol rod, which could not be compared to the current device or conventional devices. The data from this study shows higher overall load transfer, with minimal differences in rigid and PEEK rods. Their data show a stark improvement in anterior column load-sharing for PEEK rods which was not observed in the present study.

The kinematics of the two semirigid systems investigated was very similar. There was not much difference in the response of the spine to either device, with or without interbody fusion—in terms of motion output. Nevertheless, as mentioned, there were clear differences in load-sharing. Rigid and semirigid PEEK instrumentation formed concentrated pressures on the area of first contact, while the semirigid dynamic stabilization system redistributed load away from the initial contact point. Ultimately with the PDS device, the total force through the spacer was larger, but the maximum pressures were reduced. PEEK rods appear to provide some benefit in terms of total force transmission through the interbody space, albeit statistically insignificant, but like rigid rods suffer from very nonuniform regions of loading across the spacer area.

## 5. Conclusion

Load-sharing of the spinal column is an important biomechanical factor which may directly affect a patient's pain level, fusion success, and future disease progression, following spine surgery. In this study, load-sharing and regional load distribution of the interbody area were compared between rigid rods, semirigid PEEK rods, and semirigid dynamic posterior instrumentation with flexion-extension dampening materials. Despite similarities in motion characteristics between PEEK rods and PDS systems, the overall load-sharing was highest for the PDS device, with marginal differences between rigid and semirigid PEEK instrumentation. The PDS system reduced regional pressure gradients and was more uniform in the anterior, posterior, left, and right interbody spaces when compared to the other instrumentation types. The semirigid PEEK rods had the least uniform distribution in contact pressure. The outcomes reported here are encouraging for the use of PDS devices, but more clinical evaluation is needed to understand how load-sharing properties relate to clinical outcomes.
